# Magnesium Deprivation Potentiates Human Mesenchymal Stem Cell Transcriptional Remodeling

**DOI:** 10.3390/ijms19051410

**Published:** 2018-05-09

**Authors:** Azzurra Sargenti, Sara Castiglioni, Elena Olivi, Francesca Bianchi, Alessandra Cazzaniga, Giovanna Farruggia, Concettina Cappadone, Lucia Merolle, Emil Malucelli, Carlo Ventura, Jeanette A. M. Maier, Stefano Iotti

**Affiliations:** 1 Department of Pharmacy and Biotechnology, University of Bologna, 40127 Bologna, Italy; azzurra.sargenti@unibo.it (A.S.); giovanna.farruggia@unibo.it (G.F.); concettina.cappadone@unibo.it (C.C.); emil.malucelli@unibo.it (E.M.); 2 Department of Biomedical and Clinical Sciences ‘L. Sacco’, University of Milan, 20157 Milan, Italy; sara.castiglioni@unimi.it (S.C.); alessandra.cazzaniga@unimi.it (A.C.); jeanette.maier@unimi.it (J.A.M.M.); 3 GUNA—ATTRE (Advanced Therapies and Tissue Regeneration), Innovation Accelerator at CNR, Via Gobetti 101, 40129 Bologna, Italy; elecorte82@gmail.com (E.O.); carlo.ventura@unibo.it (C.V.); 4 National Institute of Biostructures and Biosystems (NIBB), 00136 Rome, Italy; francibi@alice.it; 5 Transfusion Medicine Unit, Azienda Usl di Reggio Emilia-IRCCS, 42123 Reggio Emilia, Italy; lucia.merolle@ausl.re.it; 6 National Laboratory of Molecular Biology and Stem Cell Engineering—Eldor Lab, Innovation Accelerator at CNR, Via Gobetti 101, 40129 Bologna, Italy

**Keywords:** magnesium, human bone marrow mesenchymal stem cells, human adipose-derived mesenchymal stem cells, osteogenic differentiation, transcriptional remodeling

## Abstract

Magnesium plays a pivotal role in energy metabolism and in the control of cell growth. While magnesium deprivation clearly shapes the behavior of normal and neoplastic cells, little is known on the role of this element in cell differentiation. Here we show that magnesium deficiency increases the transcription of multipotency markers and tissue-specific transcription factors in human adipose-derived mesenchymal stem cells exposed to a mixture of natural molecules, i.e., hyaluronic, butyric and retinoid acids, which tunes differentiation. We also demonstrate that magnesium deficiency accelerates the osteogenic differentiation of human bone marrow-derived mesenchymal stem cells. We argue that magnesium deprivation generates a stressful condition that modulates stem cell plasticity and differentiation potential. These studies indicate that it is possible to remodel transcription in mesenchymal stem cells by lowering extracellular magnesium without the need for genetic manipulation, thus offering new hints for regenerative medicine applications.

## 1. Introduction

Magnesium (Mg) is essential for life and health, since it is implicated in all metabolic and biochemical processes in the cell, including energy metabolism, DNA duplication, RNA transcription, protein synthesis and redox reactions [1]. Mg also acts as an intracellular second messenger and as a natural calcium antagonist [2,3]. A large amount of data shows a compelling role for Mg in the maintenance of cellular physiology and in the control of cell proliferation [3], because Mg contributes to the regulation of the cell cycle. Initially, Mg is mandatory for the activation of the kinases that transduce the mitotic signal from plasma membrane receptors to the nucleus. Next, Mg is required to sustain protein synthesis and to replicate DNA [3]. Accordingly, intracellular Mg increases fractionally in the G1 and S phase of the cell cycle. In the late stages, Mg has a role in the assembly of the mitotic spindle [4]. On these bases, it is not surprising that low concentrations of extracellular Mg inhibit the growth of cultured mammalian cells by decreasing the number of cells in the S phase and promoting their accumulation mainly in the G0/G1 and also in G2/M phases of the cell cycle [1]. In Mg-deficient conditions, growth inhibition also results from aberrant mitochondrial function with the consequent loss of control over cellular energy turnover [5], hence generating energetic stress [6]. In agreement with these findings, chemical or genetic inhibition of Mg transporters and channels constrains cell growth, and this can be overridden by Mg supplementation [7]. While the link between Mg and cell growth has been deeply investigated, comparatively little is known on the role of this element in cell differentiation. A few studies suggest that extracellular Mg differently affects cell differentiation [7,8,9,10]. Recently, intriguing results were obtained in human embryonic stem cells [11]. Mg withdrawal induced by mesendogen, an inhibitor of TRPM6/TRPM7 (the principal ion channels responsible for cellular Mg import), promotes mesoderm and definitive endoderm differentiation [11].

It is emerging that different kinds of cellular stress enhance or modulate cell differentiation [12,13,14,15]. The concept that stress has a fundamental role in cell plasticity and cell reprogramming has been recently reviewed [16]. It is feasible to hypothesize that stress primes the cells to a phase of readiness to cope with a hostile or simply unusual environment.

Since (i) Mg deprivation is associated with energetic and oxidative stress in several cell types and tissues [6,17,18], and (ii) redox regulation is implicated in various differentiation and de-differentiation processes [14], we reasoned that Mg withdrawal might reprogram and modulate the differentiation of mesenchymal stem cells. In this light, a proper tuning of Mg homeostasis can be viewed as a novel tool to chemically manipulate stem cell fate without the need of viral vector mediated gene transfer technologies or the use of cumbersome chemistry for the development of synthetic differentiating agents.

In this study, the rationale was to investigate the effects of the stress generated by Mg deprivation both on cell reprogramming and terminal cell differentiation. These are distinct and complementary processes, which require the use of different experimental models and different protocols. To accomplish this task, we have selected two different mesenchymal stem cell models, i.e., adipose-derived mesenchymal stem cells (AD-MSCs) and bone marrow mesenchymal stem cells (BM-MSCs), whose reprogramming and differentiation protocols are well established and standardized. Indeed, while both cell types are osteogenic, BM-MSCs have been extensively used to investigate osteoblastic differentiation and seem to perform better than AD-MSCs [19], while AD-MSCs have been widely used as multilineage cell model, partly thanks to the minimally invasive harvest of the adipose tissue and the ease of obtaining large amounts of cells [20,21,22].

AD-MSCs were cultured in normal or Mg-deficient media in the presence of a reprogramming medium composed of a mixture of hyaluronic, butyric and retinoic acids (hereafter referred to as RM) [23,24], a cocktail which tunes differentiation potential. Indeed, previous work has demonstrated that this mixture commits human amniotic fluid stem cells toward cardiac and angiogenic phenotype [24]. We then assessed the expression of the multipotency gene Nanog Homeobox (*NANOG*) and tissue-restricted transcription factors, such as GATA Binding Protein 4 (*GATA-4*), NKX2 Homeobox 5 (*NKX-2.5*), Hepatocyte Growth Factor (*HGF*), Kinase Insert Domain Receptor (*KDR*), Neurogenin (*NEUROG*). *GATA-4* and *NKX-2.5* are two key regulators of cardiogenic commitment. In particular, *NKX-2.5* is the earliest marker of the cardiac lineage [25]. *HGF* and *KDR* are involved in the orchestration of vasculogenesis and proper capillary formation [26,27]. *NEUROG* is a marker of neurogenic commitment [28], while *NANOG* is involved in the maintenance of stem cell pluripotency [29,30].

We also examined the impact of Mg deprivation on the osteogenic differentiation of BM-MSCs treated with vitamin D and glycerolphosphate [31]. We evaluated the expression of transcription factors required for osteogenesis, as well as the deposition of extracellular calcium, since the formation of a mineralized extracellular matrix is a hallmark of osteogenic differentiation.

## 2. Results

### 2.1. Mg and the Transcriptional Remodeling of Adipose-Derived Mesenchymal Stem Cells (AD-MSCs)

**Figure 1:**
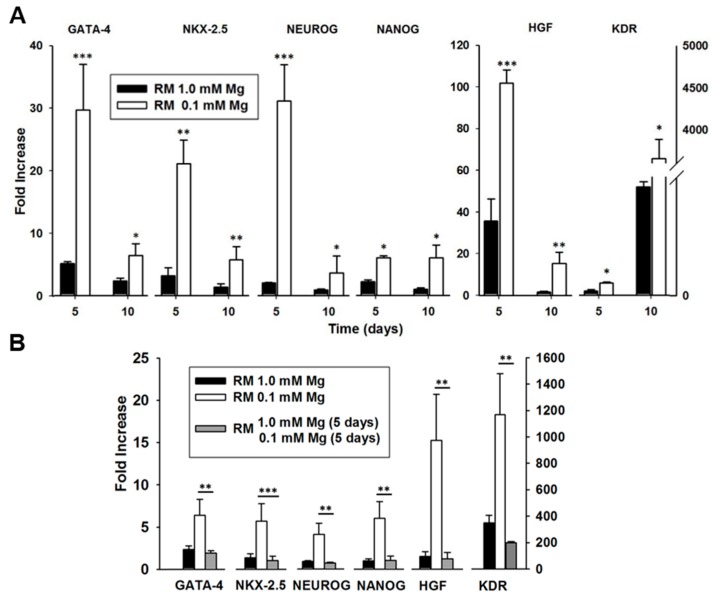
Effect of Mg withdrawal and re-supplementation on gene expression in adipose-derived mesenchymal stem cells (AD-MSCs). (**A**) Expression of multilineage markers in AD-MSCs treated in reprogramming medium (RM 1.0 mM Mg, black bar), or in Mg-deficient medium (RM 0.1 mM Mg, white bar). From the left: *GATA-4*, *NKX-2.5*, *NEUROG*, *NANOG*, *HGF*, *KDR*. All the values were normalized with respect to their untreated controls. * *p* < 0.05, ** *p* < 0.01, *** *p* < 0.001. (**B**) Expression of *GATA-4*, *NKX-2.5*, *HGF*, *NEUROG*, *NANOG* and *KDR* in cells cultured in complete RM (black bar) or in Mg-deficient medium (white bar) for 10 days. Some samples were kept in Mg-deficient medium for 5 days and then supplemented with 1 mM Mg for additional 5 days (grey bar). All the values were normalized with respect to their untreated controls (i.e., without the reprogramming cocktail). The results are the mean of three experiments carried out in triplicate. ** *p* < 0.01, *** *p* < 0.001.

AD-MSCs were cultured for 5 and 10 days in normal or Mg-deficient medium in the absence or in the presence of a cocktail containing hyaluronic, butyric and retinoic acids (reprogramming medium, RM) [23,24]. We examined gene expression of a panel of markers representing the multilineage potential of these cells, such as *GATA-4*, *NKX-2.5*, *HGF*, *KDR*, *NEUROG* and *NANOG*. By Real-time PCR we found that the expression of the aforementioned markers is significantly enhanced in RM-treated cells under Mg-deficiency compared to RM-treated controls after 5 and 10 days from the beginning of the experiment (Figure 1A).

To further dissect the involvement of Mg in the modulation of gene expression in AD-MSCs, we examined the levels of these transcripts in RM-treated cells cultured in Mg-deficient medium for 5 days and then supplemented with Mg to reach the physiologic concentration of 1 mM. We found that the Mg supplementation decreased the expression of all the genes to the same level of samples cultured in complete medium (Figure 1B), thus demonstrating that the enhancement of the reprogramming markers induced by Mg deficiency is fully reversible. Based on these observations, the transcriptional remodeling of Mg-deprived cells cultured in RM can be viewed as a response to the dramatic, non-physiological external trigger represented by Mg deficiency.

**Figure 2:**
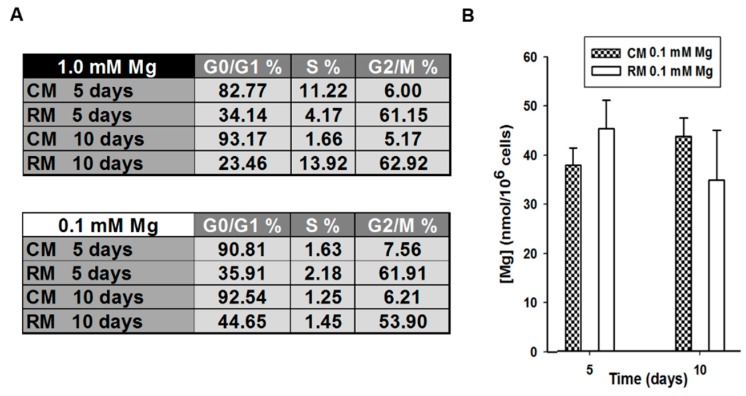
Effects of Mg withdrawal on cell cycle distribution and intracellular Mg concentration in adipose-derived mesenchymal stem cells (AD-MSCs). (**A**) Cell cycle distribution of AD-MSCs cultured in reprogramming medium (RM) or control medium (CM) at 5 and 10 days in physiological concentrations of Mg (upper table) or in Mg-deficient medium (lower table). The results are the mean of three experiments, carried out in triplicate. (**B**) Total Mg concentration was measured in treated (RM 0.1 mM Mg) and untreated (CM 0.1 mM Mg) AD-MSCs after 5 and 10 days in Mg-deficient medium. Measurements were carried out in sonicated sample by using the fluorescent probe DCHQ5.

The study of the mechanisms that govern self-renewal and lineage specification are still poorly explored. Because cell cycle position seems to influence the response to differentiation agents [32], we determined cell cycle profile by flow cytometry in control and stimulated AD-MSCs cultured in Mg-deficient media for 5 and 10 days. Interestingly, we observed a remarkable accumulation of cells in the G2/M phase in treated cells at all times tested (Figure 2A, lower table). Moreover, both control and stimulated Mg-deprived AD-MSCs showed the same intracellular total Mg content (Figure 2B). This suggests that the block of the cell cycle at G2/M phase is induced by the RM rather than Mg deprivation (Figure 2A, lower table), since RM-exposed cells showed an accumulation in the G2/M phase of the cell cycle also in complete medium (Figure 2A, upper table).

No alteration in the production of reactive oxygen species (ROS) was detected in AD-MSCs cultured in Mg-deficient conditions (Figure S1).

### 2.2. Mg Transcriptional Remodeling and Osteogenic Differentiation of Bone Marrow Mesenchymal Stem Cells (BM-MSCs)

**Figure 3:**
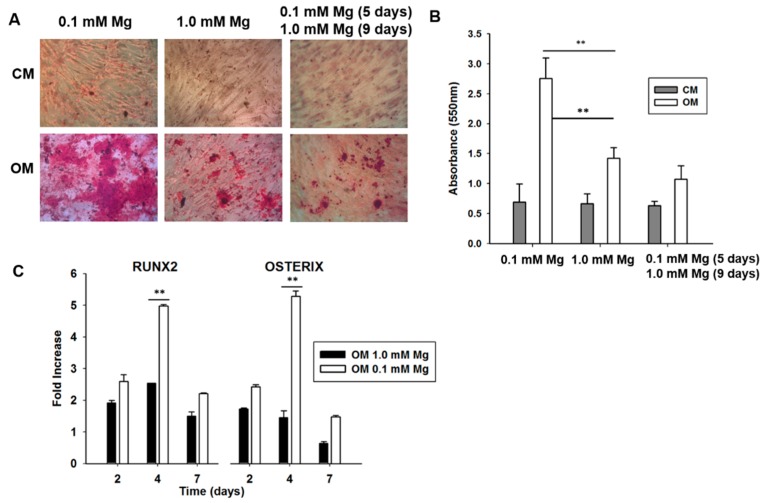
Effect of Mg withdrawal and re-supplementation in differentiating bone marrow mesenchymal stem cells (BM-MSCs). (**A**) BM-MSCs were cultured in 1 mM Mg (middle panel) or in Mg-deprived medium (left panel) and exposed to control medium (CM) or osteogenic medium (OM). BM-MSCs were cultured in Mg-deficient medium for 5 days and in 1 mM Mg for the following 9 days. After 14 days we evaluated the deposition of calcium phosphate in the extracellular matrix by Alizarin Red S staining. Photographs were taken at 10× magnification. (**B**) The absorbance of Alizarin Red S staining was measured at 550 nm. ** *p* < 0.01. (**C**) BM-MSCs were cultured in 1 mM Mg or in Mg-deprived medium and exposed to CM or OM for 2, 4 and 7 days. Real-time PCR was performed three times, carried out in triplicate, on RNA extracted using primers designed on *RUNX2* and *Osterix* (*OSX*) sequence. All the values were normalized with respect to their controls cultured in CM. ** *p* < 0.01.

We then turned our attention to BM-MSCs, which are capable of differentiating into osteoblasts, chondrocytes and adipocytes in response to specific environmental clues [33]. In particular, we tested whether and how Mg deprivation impacts on the differentiation into osteoblasts of BM-MSCs. Confluent cells were induced to differentiate in normal or Mg-deficient medium containing vitamin D, glycerolphosphate and ascorbic acid. Initially, we evaluated the deposition of calcified extracellular matrix after 14 days of induction. By Alizarin Red S staining, we found that the degree of calcium deposition was higher in BM-MSCs induced to differentiate in Mg-deficient medium, thus indicating that Mg deprivation enhanced the response to the osteogenic cocktail (Figure 3A,B). It is noteworthy that similar results were obtained in two additional healthy male donors (Figure S2), and also when differentiation was induced by dexamethasone instead of vitamin D (Figure S3). We then investigated whether the stimulatory effect of Mg deficiency on osteogenic differentiation was reversible. For this purpose, we cultured BM-MSCs in Mg-deficient medium for 5 days and then added Mg to reach its physiological concentration for the following 9 days. As shown in Figure 3A,B, the deposition of calcified matrix is comparable in control BM-MSCs and in cells deprived of Mg for 5 days and then restored to physiologic concentration. We argue that, once the early genetic program for differentiation is activated in Mg-deficient cells, the return to 1 mM restores the normal physiologic response to the osteogenic cocktail.

Next, we evaluated the expression of two early markers of osteogenic differentiation, i.e., *RUNX2* and *Osterix* (*OSX*), transcription factors essential for osteoblast differentiation and bone formation during embryonic development, but crucial also for postnatal bone growth and homeostasis. In particular, since no ossification occurs in *RUNX2*^−/−^ mice, *RUNX2* is considered the master regulator of osteogenesis, while *OSX* is necessary to activate the early stages of osteogenesis but is not sufficient for complete differentiation. By Real-time PCR on samples collected at different times after induction, we found that Mg deficiency sensitizes BM-MSCs to the osteogenic cocktail (Figure 3C). Indeed, at day 4, the differentiation cocktail induced both *RUNX2* and *OSX* more markedly in Mg-deficient cells than in their controls in 1 mM Mg. Figure S4 shows that the induction of *COL1A1*, essential for the progression of differentiation, and *BGLAP*, a non-collagenous component of bone extracellular matrix, is greater in Mg-deficient BM-MSCs.

**Figure 4:**
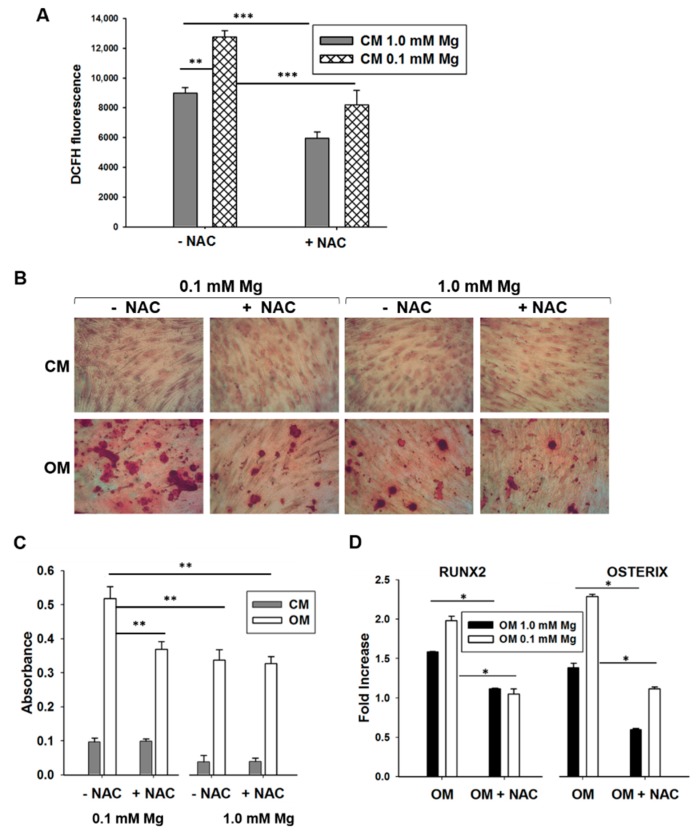
Relationship between Mg withdrawal and reactive oxygen species (ROS) generation. (**A**) Bone marrow mesenchymal stem cells (BM-MSCs) were cultured in 1 mM Mg or in Mg-deprived medium and exposed to control medium (CM). The cells were then treated with *N*-acetylcysteine (NAC, 1 mM) for 24 h. ROS generation was measured. Data are shown as the mean of three separate experiments ± standard deviation. ** *p* < 0.01, *** *p* < 0.001. (**B**) BM-MSCs were cultured in 1 mM Mg or in Mg-deficient medium and exposed to CM or to osteogenic medium (OM). The cells were then treated with NAC (1 mM) for 14 days. Alizarin Red S staining was performed, and photographs were taken at 10× magnification. (**C**) The absorbance of Alizarin Red S staining was measured at 550 nm. ** *p* < 0.01. (**D**) BM-MSCs were cultured in 1 mM Mg or in Mg-deficient medium and exposed to CM or OM. The cells were then treated with NAC for 4 days. Real-time PCR was performed three times in triplicate on RNA extracted using primers designed on *RUNX2* and *OSX* sequence. All the values were normalized with respect to their controls cultured in CM. The results are the mean of three experiments in triplicates. * *p* < 0.05.

It is known that (i) culture in low extracellular Mg is linked to an increased generation of ROS in many cell types [18]; and (ii) the generation of ROS is needed for osteogenic differentiation of BM-MSCs. Accordingly, in BM-MSCs exposed to Mg-deficient medium, we reproducibly found a 30% increase of ROS production, which was blocked by the synthetic antioxidant *N*-acetylcysteine (NAC, 1 mM) (Figure 4A). On these bases, it is not surprising that NAC prevents the enhancement of BM-MSC differentiation by low extracellular Mg (Figure 4B–D). Indeed, in the presence of NAC, no significant differences occurred in the expression of osteogenic markers and in extracellular calcium deposition in BM-MSCs induced to differentiate in normal or Mg-deficient medium (Figure 4B–D).

## 3. Discussion

We demonstrate that both adipose- and bone marrow-derived mesenchymal stem cells are sensitized to differentiation stimuli when cultured in low extracellular Mg. This effect is promptly reversible after restoring the physiologic concentration of the cation, indicating that no permanent modifications occur as a result of the deprivation of the cation in the time frame utilized in this study, as previously shown in other cell types [34].

Mg deficiency potentiates the commitment of AD-MSCs with a mixture of hyaluronic, butyric and retinoic acids toward multiple lineage, including neuronal, cardiac and vascular fates. Concomitant with the expression of lineage-restricted genes, Mg deficiency also enhances the expression of the stemness-related gene *NANOG* after exposure to the reprogramming cocktail. It is reported that *NANOG* is expressed in cultured mesenchymal stem cells, while *SOX2* and *OCT4*, which regulate the maintenance of the pluripotent state in embryonic stem cells, are not [35]. Since *NANOG* expression (i) does not associate with the proliferative and differentiative potential of MSCs, and (ii) does not pinpoint cells having stem or progenitor cell properties, *NANOG* seems to contribute to regulate cell adaption to in vitro growth conditions [36]. We hypothesize that the induction of *NANOG* by Mg deficiency has a role in maintaining the cells viable and ready to respond to specific stimuli. This is a relevant issue, because low survival rates are the major obstacle to a broad clinical use of MSCs.

The mechanisms underlying the effect of Mg deficiency in implementing the response to the reprogramming medium of AD-MSCs remain elusive. Initially, we reasoned that low Mg could influence cell cycle position, which is known to affect the response to differentiation agents in MSCs [32]. Indeed, pluripotent stem cells are more responsive to specification clues in the G1 phase of the cell cycle [32,37], while apigenin and nocodazole induce cell differentiation in rat neural cells and in Caco-2 cells, respectively, during the G2/M phase [38,39]. Moreover, human embryonic stem cells in the early stages of endomesodermal differentiation require a pause in the G2 phase of the cell cycle [40].

In our experimental model, we found that the transcriptional remodeling of AD-MSCs in response to the reprogramming cocktail is strictly related to the block of the cell cycle at the G2/M phase and totally independent from extracellular Mg availability.

Then, since (i) low extracellular Mg induces the synthesis of ROS in various cell types [41], and (ii) treatment with low concentrations of H_2_O_2_ fortifies MSCs against oxidative damage [42], we evaluated ROS production in AD- and BM-MSCs. We found an increased accumulation of ROS only in BM-MSCs cultured in low vs. their controls in physiologic Mg. The different behavior of AD- and BM-MSCs might be ascribed to the high anti-oxidant arsenal of AD-MSCs. Indeed, these cells are used to accelerate wound healing because of their anti-oxidant effects [43]. In the case of BM-MSCs, oxidative stress blunts osteoblast differentiation, but osteogenic differentiation is partly ROS dependent. Indeed, regulated ROS production is increasingly recognized as fundamental in promoting essential signaling pathways that regulate stem cell proliferation, survival and differentiation [44,45]. Appropriate levels of ROS are required for the homing of hematopoietic stem cells to the bone marrow after transplantation [46]. Moreover, an increase of endogenous ROS regulates self-renewal and neurogenesis of neural stem cells [47]. On these bases, we hypothesize that Mg deficiency reversibly accelerates BM-MSC differentiation in osteoblasts, partly through a modest increase of ROS.

Most cells sense Mg deficiency as a stressful condition and, consequently, activate adaptative responses [18]. As an example, endothelial cells cultured under Mg-deficient conditions upregulate stress proteins and this event modulates the functions of the cells [41]. In the case of MSCs, we hypothesize that the cells react to Mg deprivation, which might threaten their integrity, by remodeling their transcription as an attempt to escape damage and to be ready for regeneration upon return to physiologic conditions. More studies are necessary to understand the mechanisms underlying the effects of Mg deficiency on stem cell differentiation. An interesting aspect to explore is whether Mg deprivation impacts on exosomes’ release and profile. Indeed, exosomes, 30–100 nm lipid vesicles that serve as important mediators of intercellular communication [48], have been shown to regulate osteoblast differentiation [49]. To the best of our knowledge, there is only one report linking low Mg to exosomes, showing that Mg deficiency reduces the release of exosomes from placental microvascular endothelial cells [50].

## 4. Materials and Methods

### 4.1. Isolation and Culture of Human Mesenchymal Stem Cells

According to the policies approved by the Institutional Review Boards for Human Studies local ethical committees, all tissue samples were obtained after oral informed consent from all subjects and all experiments were performed in accordance with relevant guidelines and regulations approved by the Ethics Committee of the University of Bologna (prot. no. 31335-X/10, 22 July 2011). Human subcutaneous adipose tissue samples were obtained from lipoaspiration procedures and processed mechanically by using the Lipogems device, as previously described [51]. Lipogems product was cultured, until the AD-MSCs began to adhere and grow on the flasks [52]. These cells were characterized for the expression of specific surface markers (Table S1), and the potential to differentiate along the adipogenic, chondrogenic, and osteogenic lineages was previously assayed [51]. AD-MSCs were obtained from three female donors with an average age of 44.0 ± 7.2 years.

Cells were cultured in minimum essential medium–α modification (α-MEM, Lonza, Walkersville, MD, USA) supplemented with 10% heat-inactivated fetal bovine serum (FBS, Euroclone, Milan, Italy), 1% penicillin/streptomycin solution (10,000 U/mL penicillin, 10,000 mg/L streptomycin, Euroclone), 2 mM l-glutamine (Lonza). To study the transcriptional remodeling of these cells, twenty-four hours after seeding, they were cultured in absence (control medium, CM) or presence (reprogramming medium, RM) [23] of a mixture containing 10–20 kDa hyaluronic (2 mg/mL, Lifecore Biomedical, Chaska, MN, USA, cat. no. HA10K), butyric (5 mM, Sigma-Aldrich, St. Louis, MO, USA, cat. no. B5887) and retinoic acid (1 μM, Sigma-Aldrich, cat. no. R2625). Mg-free minimum essential medium (MEM) was purchased as customized medium (Invitrogen, Carlsbad, CA, USA) and supplemented with MgCl_2_ (1 mM, Sigma-Aldrich, cat. no. M8266). In some experiments, the cells were cultured for 5 days in Mg-deficient medium, and then MgCl_2_ was added to reach the physiologic concentration of 1 mM for additional 5 days.

BM-MSCs were isolated from human bone marrow withdrawn from bilateral punctures of the posterior iliac crests of three healthy male volunteers (age 35–45), after obtaining oral informed consent from all the subjects, and tested for purity by flow cytometry at the Policlinico in Milan according to institutional guidelines and regulations approved by the IRCCS Policlinico and Università degli Studi di Milano [53]. These cells were characterized for surface markers (Table S1), and were demonstrated to undergo osteogenic and adipogenic differentiation in response to specific stimuli [31]. BM-MSCs were cultured in Dulbecco’s modified Eagle’s medium (DMEM, Lonza) with 1000 mg/L glucose, 10% FBS and 2 mM l-glutamine (CM).

In some experiments, BM-MSCs were cultured in the presence of *N*-acetylcysteine (NAC, 1 mM, Sigma-Aldrich, cat. no. A7250). To induce osteogenic differentiation, BM-MSCs were seeded in 6-well or 96-well plates. Once the cells were confluent, an osteogenic induction cocktail was added to the medium (osteogenic medium, OM). The osteogenic cocktail contains 2 × 10^−8^ M 1α,25-Dihydroxyvitamin D_3_, 10 mM β-glycerolphosphate and 0.05 mM ascorbic acid (Sigma-Aldrich) [31]. To investigate calcium deposition by BM-MSCs, the cells were rinsed with PBS, fixed (70% ethanol, 1 h) and stained for 10 min with 2% Alizarin Red S (pH 4.2, Sigma-Aldrich) [31]. The experiment was repeated three times in triplicate. Photographs were taken at 10× magnification. Alizarin Red S staining was released from the cell matrix by incubation in 10% cetylpyridinium chloride in 10 mM sodium phosphate (pH 7.0), for 15 min and the absorbance measured at 550 nm.

All the cells were maintained at 37 °C and 5% CO_2_ in a humidified atmosphere.

### 4.2. Gene Expression Analysis

After 5 and 10 days of treatment, total RNA was extracted using RNeasy Microkit (Qiagen, Hilden, Germany), and 1 μg was reverse-transcribed into cDNA in a 21-μL reaction volume with SuperScriptTM III Reverse Transcriptase (Life Technologies, Carlsbad, CA, USA). Real-time PCR was performed as previously described [24,31]. The experiment was performed 3 times in triplicate and each sample was run in triplicate. The average threshold cycle (*C*t) values were used for calculations. Obtained *C*t values were normalized to glyceraldehyde-3-phosphate dehydrogenase (*GAPDH*) transcript levels. Relative quantification of mRNA expression was calculated with the comparative *C*t method using the “delta-delta method” for comparing relative expression results between treatments [54]. The values of the transcripts were normalized with respect to their untreated controls.

See Table S2 for AD-MSC primer sequences.

For BM-MSC Real-time PCR, TaqMan Gene Expression Assays (Life Technologies) were used: Hs00231692_m1 (*RUNX2*), Hs01866874_s1 (*OSX*), Hs00164004_m1 (*COL1A1*), Hs01587814_g1 (*BGLAP*), Hs99999905_m1 (*GAPDH*).

### 4.3. Cell Cycle Analysis

Human adipose-derived MSCs were detached with 0.1% trypsin, 0.02% EDTA, washed twice in DPBS and centrifuged. The pellet was suspended in 0.01% nonidet P-40, 10 mg/mL RNase, 0.1% sodium citrate and 50 μg/mL propidium iodide (PI), for 30 min at room temperature in the dark. Propidium iodide fluorescence was analyzed using a Bryte HS flow cytometer (Bio-Rad, Hercules, CA, USA) equipped with Hg lamp and analyzed with ModFit 4(Verity Software House, Topsham, ME, USA) software.

### 4.4. Quantification of Total Cell Mg by Spectrofluorimetric Assay

Total Mg content was assessed on sonicated cell samples of AD-MSCs by the fluorescent dye DCHQ5 as reported as previously described [55,56].

### 4.5. Reactive Oxygen Species Evaluation

Intracellular oxidative stress was quantified using 2′-7′-dichlorofluorescein diacetate (DCFH, Sigma-Aldrich, cat. no. 35845). Cells were seeded into black bottomed 96-well plates (Greiner bio-one, Frickenhausen, Germany), cultured in medium containing 1 mM Mg or in Mg-deficient medium. Some samples were treated with NAC 1 mM for 24 h. The cells were then washed with PBS and exposed to DCFH (20 µM). The rate of intracellular oxidative stress was evaluated by monitoring the emission at 529 nm of the DCFH dye using GloMax^®^-Multi Detection System (Promega, Madison, WI, USA). Three independent experiments were performed. Data are shown as the mean ± standard deviation.

### 4.6. Statistical Analysis

Differences of each experimental condition were evaluated by Student’s *t* test and set as follows: * *p* < 0.05, ** *p* <0.01, *** *p* < 0.001.

## 5. Conclusions

These results offer new perspectives in stem cell research and in regenerative medicine showing that Mg withdrawal could be exploited as a potentiating tool to enhance cell reprogramming and differentiation.

## Supplementary Materials

Supplementary materials can be found at http://www.mdpi.com/1422-0067/19/5/1410/s1.

## Abbreviations

AD-MSCs	Adipose-Derived Mesenchymal Stem Cells
BM-MSCs	Bone Marrow Mesenchymal Stem Cells
NANOG	Nanog Homeobox
GATA-4	GATA Binding Protein 4
NKX-2.5	NKX2 Homeobox 5
HGF	Hepatocyte Growth Factor
KDR	Kinase Insert Domain Receptor
NEUROG	Neurogenin
ROS	Reactive Oxygen Species
OSX	Osterix
NAC	*N*-acetylcysteine
CM	Control Medium
RM	Reprogramming Medium
OM	Osteogenic Medium
GAPDH	Glyceraldehyde-3-Phosphate Dehydrogenase
PI	Propidium Iodide
DCFH	2′-7′-Dichlorofluorescein Diacetate
