# Supplementary material for: Magnesium Deprivation Potentiates Human Mesenchymal Stem Cell Transcriptional Remodeling
Source: Int J Mol Sci. 2018 May 9;19(5):1410. doi: 10.3390/ijms19051410 (PMC5983826; doi:10.3390/ijms19051410)
Supplement: Supplementary file 1 [file ijms-19-01410-s001.pdf]

Supplementary

Magnesium deprivation potentiates human mesenchymal stem cell transcriptional remodeling

Azzurra Sargenti [1,†], Sara Castiglioni [2,†], Elena Olivi [3,4,†], Francesca Bianchi [4], Alessandra Cazzaniga [2], Giovanna Farruggia [1,4], Concettina Cappadone [1], Lucia Merolle [5], Emil Malucelli [1], Carlo Ventura [3,4,6], Jeanette A.M. Maier [2] and Stefano Iotti [1,4,*]

[1] Department of Pharmacy and Biotechnology, University of Bologna, 40127 Bologna, Italy; azzurra.sargenti@unibo.it (A.S.); giovanna.farruggia@unibo.it (G.F.); concettina.cappadone@unibo.it (C.C.); emil.malucelli@unibo.it (E.M.); stefano.iotti@unibo.it (S.I.)
[2] Department of Biomedical and Clinical Sciences 'L. Sacco', University of Milan, 20157 Milan, Italy; sara.castiglioni@unimi.it (S.C.); alessandra.cazzaniga@unimi.it (A.C.); jeanette.maier@unimi.it (J.A.M.M.)
[3] GUNA—ATTRE (Advanced Therapies and Tissue Regeneration), Innovation Accelerator at CNR, Via Gobetti 101, 40129 Bologna, Italy; elecorte82@gmail.com (E.O.); carlo.ventura@unibo.it (C.V.)
[4] National Institute of Biostructures and Biosystems (NIBB), 00136 Rome, Italy; francibi@alice.it (F.B.)
[5] Transfusion Medicine Unit, Azienda Usl di Reggio Emilia-IRCCS, 42123 Reggio Emilia, Italy; lucia.merolle@ausl.re.it (L.M.)
[6] National Laboratory of Molecular Biology and Stem Cell Engineering—Eldor Lab, Innovation Accelerator at CNR, Via Gobetti 101, 40129 Bologna, Italy.
* Correspondence: stefano.iotti@unibo.it; Tel.: +39-051-209-5413
† These authors equally contributed to this work.

Received: 13 April 2018; Accepted: 5 May 2018; Published: date

* Correspondence: stefano.iotti@unibo.it; Tel.: +39-051-209-5413

Received: date; Accepted: date; Published: date

Figure S1. No alteration in the production of reactive oxygen species (ROS) was detected in adipose-derived mesenchymal stem cells (AD-MSCs) cultured in Mg-deficient conditions.
AD-MSCs were cultured in 1 mM Mg control medium (CM) or reprogramming medium (RM), or in Mg-deprived medium (CM or RM) in the presence or absence of N-acetylcysteine (NAC, 1 mM) for 4 h. ROS generation was measured (DCFH fluorescence). Data are shown as the mean of three separate experiments ± standard deviation (Kruskal-Wallis test).

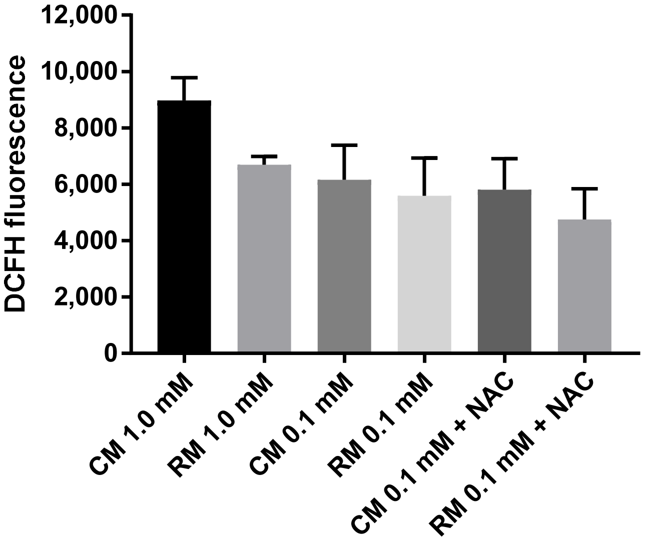

Figure S2. Effect of Mg withdrawal and re-supplementation in differentiating bone marrow mesenchymal stem cells (BM-MSCs).

BM-MSCs from three different donors were cultured in 1 mM Mg or in Mg-deficient medium (0.1 mM Mg) in the presence or in the absence of vitamin D. Results from donor 1 are reported in Fig. 3 and 4. Here we show Alizarin Red S staining for donor 2 and 3.

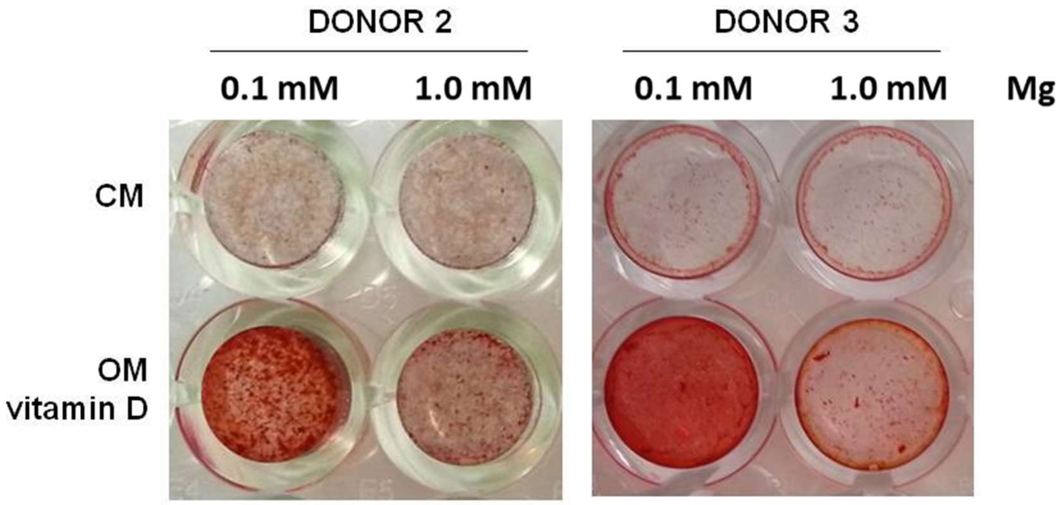

Figure S3. Effect of Mg withdrawal in differentiating bone marrow mesenchymal stem cells (BM-MSCs) induced by dexamethasone.

BM-MSCs from donor 1 were cultured in 1 mM Mg or in Mg-deficient medium (0.1 mM) in the presence or in the absence of dexamethasone (10^{-7} M). After 14 days we evaluated the deposition of calcified extracellular matrix by Alizarin Red staining. Photos were taken at 10x magnification.

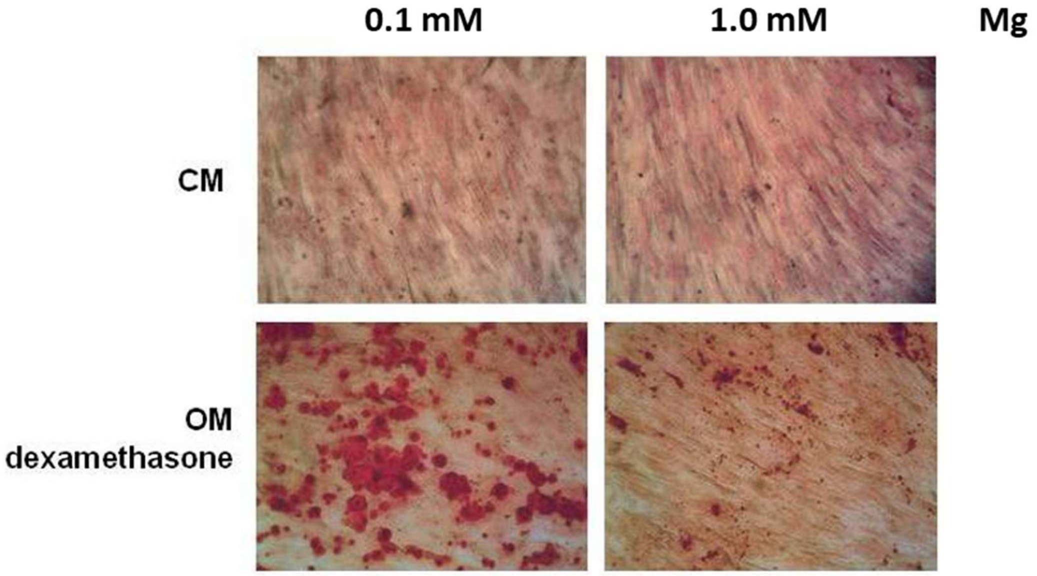

Figure S4. Effect of Mg withdrawal on gene expression in differentiating bone marrow mesenchymal stem cells (BM-MSCs).
BM-MSCs were cultured in 1 mM Mg or in Mg-deficient medium (0.1 mM) and exposed to control (CM) or osteogenic (OM) medium for 4 days. Real-time PCR was performed three times in triplicate on RNA extracted using primers designed on COL1A1 and BGLAP sequence.

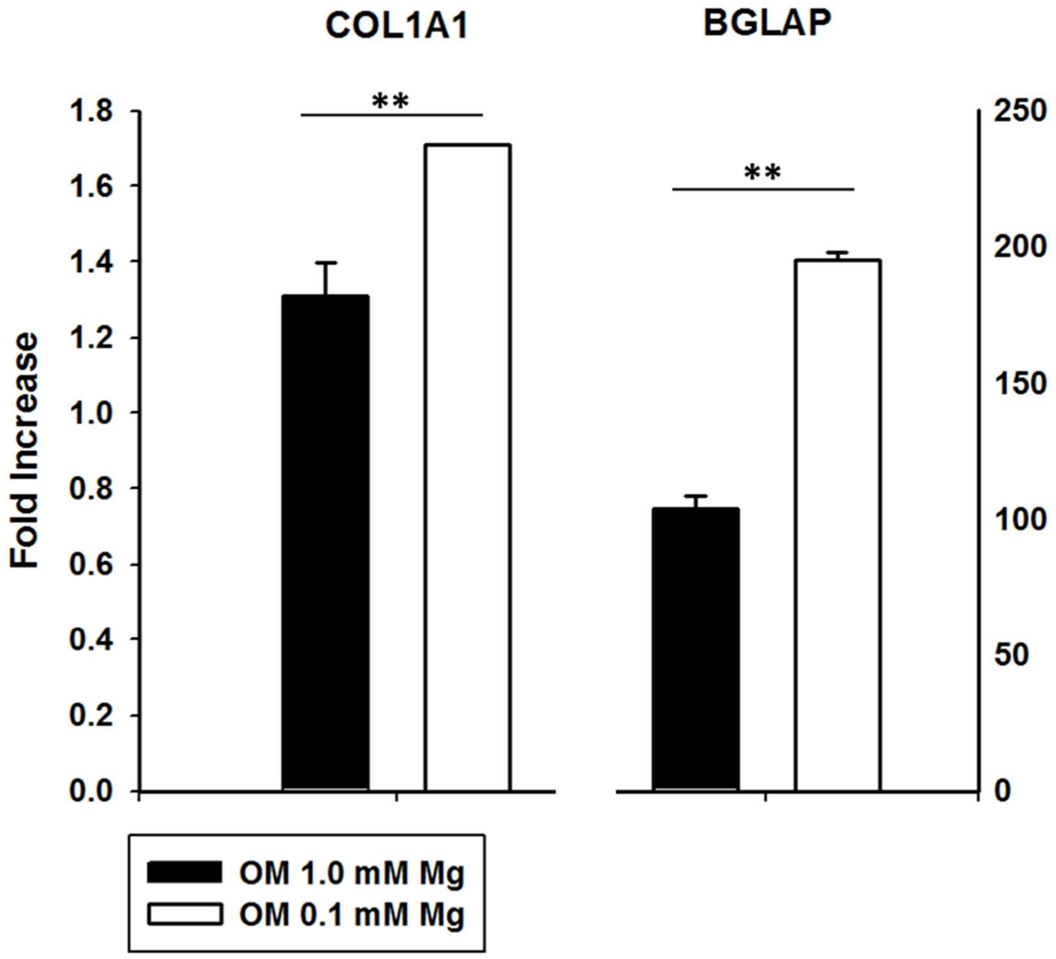

Table S1. Adipose-derived mesenchymal stem cells (AD-MSCs) and bone marrow mesenchymal stem cells (BM-MSCs) were characterized using specific antibodies by flow cytometry analysis*. Data are expressed as % ± standatd deviation.

	CD34	CD44	CD45	CD90	CD105
**AD-MSCs**	0.3 ± 0.2	97.3 ± 8.4	1.0 ± 0.3	90.1 ± 4.7	97.5 ± 2.6
**BM-MSCs**	1.2 ± 0.6	97.3 ± 8.4	1.1 ± 0.6	71.5 ± 4.4	87.3 ± 9.1

* For flow cytometry analysis, AD-MSCs and BM-MSCs were incubated with fluorescent antibodies (1 μg/106 cells) for 40 min at 4°C in the dark. After washing, cells were analyzed on a flow cytometer (FACSAria, BD Biosciences, San Jose, CA, USA) by collecting 10,000 events, and the data were analyzed using the FACSDiva Software (BD Biosciences). Anti-CD34, anti-CD44, and anti-CD45 antibodies were purchased from BD Biosciences; anti-CD90, and anti-CD105 were purchased from BioLegend (San Diego, CA, USA).

Table S2. Primer sequences for adipose-derived mesenchymal stem cells (AD-MSCs) Real-time PCR.

Gene	Primer sequence	Supplier
GAPDH	Forward: 5′-CAGCCTCAAGATCATCAGCA-3′<br>Reverse: 5′-TGTGGTCATGAGTCCTTCCA-3′	Primm
GATA-4	Forward: 5′-ACCACAGCACAGCCTCATC-3′<br>Reverse: 5′-CAGAGCGGGAAGAGGGATTT-3′	Primer Design
HGF	Forward: 5′-ATTTGGCCATGAATTTGACCT-3′<br>Reverse: 5′-ACTCCAGGGCTGACATTTGAT-3′	Primm
KDR	Forward: 5′-CTGCAAATTTGGAAACCTGTC-3′<br>Reverse: 5′-GAGCTCTGGCTACTGGTGATG-3′	Primm
NANOG	Forward: 5′-CCTTCCTCCATGGATCTGCTT-3′<br>Reverse: 5′-CTTGACCGGGACCTTGTCTTC-3′	Sigma Aldrich
NEUROG	Forward: 5′-CCGCCTTGAGACCTGCATC-3′<br>Reverse: 5′-GGCTGCCTGTTGGAGTCTG-3′	Sigma Aldrich
NKX-2.5	Forward: 5′-GCACCCACCCGTATTTATGT-3′<br>Reverse: 5′-GGGTCAACGCACTCTCTTTAA-3′	Primer Design
